# Water and 2,2,6,6-tetra­methyl­piperidine: an odd couple make a solid match

**DOI:** 10.1107/S2052252522004341

**Published:** 2022-04-27

**Authors:** Larry R. Falvello

**Affiliations:** a Instituto de Nanociencia y Materiales de Aragón (INMA), Departamento de Química Inorgánica, CSIC - Universidad de Zaragoza, Pedro Cerbuna 12, Zaragoza, 50009, Spain

**Keywords:** molecular solid, proton transport, liquid-liquid co-crystallization, 2,2,6,6-tetra­methyl­piperidine

## Abstract

Commentary is given on a paper [Butler *et al.* (2022). *IUCrJ*, **9**, 364–369] reporting the crystallization of two room-temperature liquids, water and 2,2,6,6-tetra­methyl­piperidine, to form a crystalline solid with a water-lined channel potentially capable of proton transport.

It is fair to state that in chemical research the least expected results can be the most interesting. In this issue of **IUCrJ** , Butler *et al.* (2022 ▸) report the discovery of a new molecular solid – a two-component crystal of water and 2,2,6,6-tetra­methyl­piperidine (TMP) – formed as a by-product during the course of a study on a different subject, namely the deprotonation of ferrocene derivatives using TMP as base. The new substance, which has a simple composition of TMP·2H_2_O and is stable in a vacuum manifold but is degraded when exposed to air at room temperature, has a number of features that would not have been easy to predict.

The most prominent structural feature is the formation of what Butler *et al.* describe as a water pipe. The structure, which was analyzed by single-crystal X-ray and neutron diffraction at 100 K and by neutron diffraction at 10 K, has a central channel with TMP molecules at its periphery and with water molecules forming an inner lining of the channel walls. (Such a lining can be called a bushing in mechanical jargon, but that term has not often been applied to water-based channel linings in molecular crystals.)

The overall layout of the structure, as viewed in a projection along the channel axis, can be described as a rhombic distortion of a square-prismatic proto-structure. The water aggregate has a distorted cubic motif, which is stacked along the channel axis. The presence of latent symmetry, in which the aggregate structure has more or at least different symmetry than do the individual components, dovetails with the fact that the new solid forms only for a very specific proportion of the components – the H_2_O:TMP molar ratio must be 2:1. When this odd couple of two quite different molecules do form the solid, though, they apparently do so readily, yielding centimetres-long rod-shaped crystals.

Further interest lies in the fact that both of the pure components are liquids at room temperature. This ‘liquid–liquid co-crystallization’ has been observed previously, but to my knowledge, it does not abound (Peloquin *et al.*, 2020 ▸).

I might refer to the new solid as a co-crystal to reflect the observation that H_2_O is a full structural partner and is not merely present as a dispensable solvate. However, that would risk venturing into the definition of a co-crystal, a complex subject that has been treated more fully by others (Bond, 2007 ▸; Parkin *et al.*, 2008 ▸; Aitipamula *et al.*, 2012 ▸).

Beyond the existence of the channel lined by water is the suggestive fact that there is disorder among the sites of the water hydrogen atoms. This is where the use of single-crystal neutron diffraction is critical, since with this technique even half-occupied proton sites can be characterized accurately. The TMP·2H_2_O structure has segments of hydrogen-bonded chains, which may be identified as proton wire fragments. A proton wire was the key structural element in a previous discovery by Capelli *et al.* (2013 ▸), in which it was proved by a deuterium substitution experiment that the protons along a disordered hydrogen-bonded chain of water molecules were mobile in crystals of a manganese coordination polymer.

The concept of a proton wire merits a brief description, because such a construct may exist in more crystals than those in which it has been explicitly identified as such. As described by Nagle & Morowitz (1978 ▸), a simple proton wire consists of a chain of hydrogen bonds along which proton transport can occur. The Grotthuss mechanism, a two-step "bucket-brigade" model, is invoked to explain the proton movement along the chain (Ädelroth, 2006 ▸; Cukierman, 2006 ▸).

The example reported by Capelli *et al.* (2013 ▸) has an unbounded proton wire and is an ideal example for discussion, since, as mentioned, that system was shown by experiment to transport protons. In that study, a neutron structure analysis revealed disorder between two stages of a putative Grotthuss proton transport mechanism. The disorder is shown in Fig. 1 ▸, which is based on the atomic coordinates from the neutron diffraction analysis by Capelli *et al.* (2013 ▸). The H(A) and H(B) sites are half-occupied and H(AB) are fully occupied. In Fig. 1 ▸(*a*) congener (A) is present, indicated by solid blue bonds and solid blue H atoms. The arrows indicate the concerted rotations that move protons from the H(A) to the H(B) sites in one stage of the Grotthuss mechanism. In Fig. 1 ▸(*b*), in which the H(B) sites are now occupied (solid blue bonds and atoms), the arrows represent proton hopping to nearby empty H(A) sites. This stage of the mechanism restores the original configuration shown in Fig. 1 ▸(*a*), except that each of the H(A) protons has moved to the next oxygen carrier atom along the chain.

The structured, extended water aggregate in TMP·2H_2_O, supported by its walled TMP enclosure, may be capable of at least short-range proton transport.

In addition to its structural features and possible dynamics, there is a lesson in the fact that the TMP·2H_2_O crystal forms at all. This new and interesting solid was not the target of the research that produced it. It seems that nature has not exhausted its store of surprises, and we cannot know when our next unexpected observation will turn out to be an opening to a new avenue of exploration.

## Figures and Tables

**Figure 1:**
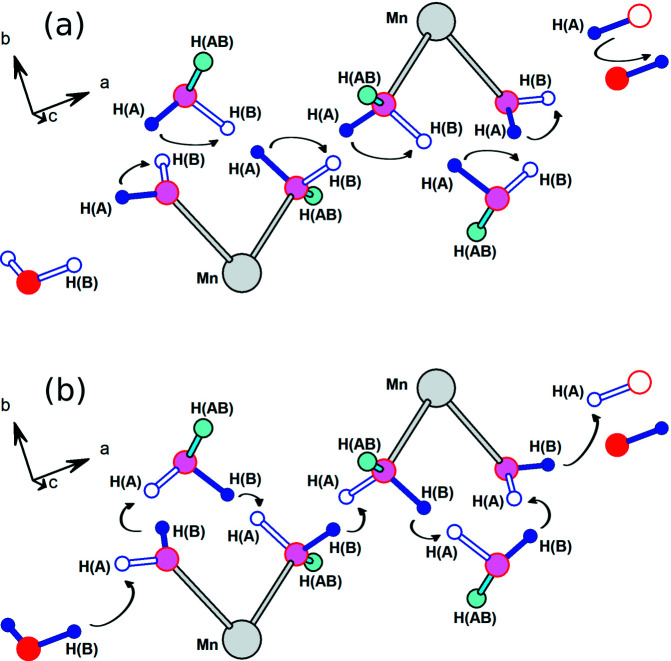
The two stages of the Grotthuss mechanism for proton transport. Atomic coordinates are from the neutron diffraction analysis of Capelli *et al.* (2013 ▸). (*a*) Reorientation of water molecules. (*b*) Proton hopping. See the text for a detailed description.
